# Limb-Girdle Muscular Dystrophy Type 2B and Morbihan Disease: A Case Report With an Atypical Presentation

**DOI:** 10.7759/cureus.93964

**Published:** 2025-10-06

**Authors:** Fernando Briceño Moya, Ana Belen Hernández Hernández, Diana Karina Delgado Carmona

**Affiliations:** 1 Internal Medicine, UMAE Hospital de Especialidades “Dr. Antonio Fraga Mouret,” Centro Médico Nacional La Raza, Instituto Mexicano del Seguro Social, Mexico City, MEX

**Keywords:** limb-girdle muscular dystrophy, morbihan disease, persistent edema, poor steroid response, progressive weakness

## Abstract

This case report presents a 38-year-old man with no significant medical history who was referred to the Internal Medicine Department due to dermatosis and muscle weakness. A multidisciplinary diagnostic approach was initiated, including laboratory, imaging, and even histopathological studies. No definitive diagnosis was obtained. During the diagnostic process, the patient's clinical course was torturous, with evidence of increasing muscle weakness and inability to walk. Due to the lack of a definitive diagnosis, empirical treatment with systemic steroids was initiated, with no clinical evidence of improvement. After ruling out a neoplastic or autoimmune cause, a molecular panel for muscular dystrophies was performed, which identified NM_003494 (DYSF_v001): c.1382T>C; p.(Ile461Thr), a clinically pathogenic and heterozygous variant. This led to the diagnosis of limb-girdle muscle dystrophy type 2B with no evidence of association with dermatosis, consistent with Morbihan disease.

Molecular diagnosis was crucial for an accurate diagnosis and thus enabled the implementation of therapeutic strategies aimed at improving the patient's quality of life.

Muscular dystrophy type 2B is a disease that still lacks specific treatment and can progress to the point of disability. The patient was fully informed about the progression and prognosis of muscular dystrophy and was referred for physical rehabilitation with the goal of delaying permanent disability. Regarding Morbihan disease, the patient received the prescribed treatment for eight months, with notable improvement in edema and dermatological lesions.

## Introduction

Muscular dystrophies are degenerative genetic disorders affecting muscle tissue. More than 40 genes are involved, giving rise to very rare variants of muscular dystrophy. According to this classification, early-onset (congenital) and late-onset (post-ambulatory) conditions are recognized, such as dysferlinopathies [1].

Dysferlinopathies are divided into Miyoshi muscular dystrophy type 1 and limb-girdle muscular dystrophy (LGMD). The latter can manifest at any age and primarily affects the muscles of the pelvic and shoulder girdles. LGMD is described as an autosomal dominant or recessive inherited disorder, with a prevalence of approximately two per 100,000 people and an incidence rate of 0.8 to 5.7 per 100,000 people, according to data from studies conducted in the United States, Asia, Europe, Africa, and Oceania. It represents the most common genetic cause of muscle weakness, with the age of onset for limb-girdle muscular dystrophy type 2 (LGMD2B) described as between 12 and 39 years [2,3].

Given the rarity and complexity of LGMD2B, it is necessary to highlight the genetic and molecular aspects of the disease. LGMD2B is described as an autosomal recessive disease (20% of all forms) due to homozygous or heterozygous mutations in the dysferlin gene (DYSF). To understand the significance of the genetic findings in this case, it is important to review the role of dysferlin, a 237 kDa protein spanning more than 150 kb of DNA, located on chromosome 2p13. This protein is normally expressed in skeletal muscle and plays an important role in muscle tissue repair. When a mutation is present, dysferlin expression is reduced or may be nonfunctional due to the mutations it may present. This impairs the defective repair of the damaged membrane, ultimately leading to muscular dystrophy. Dysferlinopathies present with atrophy and weakness of the gastrocnemius and/or tibialis anterior muscles [4,5]. Most dysferlinopathies are described as homozygous variants, so a history of consanguinity should be assessed as an important factor in the clinical history [6].

Data on the course of LGMD are limited. There are reports where, in the early stages, patients may present with an abnormal gait, difficulty walking or running, fatigue when walking long distances, and difficulty climbing stairs. As the disease progresses, this can even lead to loss of ambulation and wheelchair dependence [7].

A proper diagnosis requires a complete clinical history, including age at onset, family history, and progression of muscle weakness and atrophy. Additional studies include elevated serum creatine kinase (CK) levels, electromyography (EMG), which generally shows abnormal muscle function, magnetic resonance imaging (MRI), and genetic testing [8,9].

Regarding Morbihan disease, we can highlight the rarity of the disease and mention it as a diagnosis of exclusion in the approach to persistent facial edema.

In Morbihan disease, also known as persistent solid facial edema, solid facial lymphedema, or lymphedema rosacea, patients present with compatible dermatological lesions such as areas of erythema, papules, persistent diffuse edema, telangiectasias, pustules, and nodules located exclusively on the forehead, glabella, eyelids, and cheeks. It affects Caucasian adults of both sexes [10].

Within the diagnostic approach, other differential diagnoses should be considered, including autoimmune diseases, thyroid disorders, angioedema, sarcoidosis, chronic dermatitis, and even neoplastic diseases.

Histopathology and laboratory tests, including antinuclear antibodies (ANA) and CK, can help exclude systemic lupus erythematosus and dermatomyositis [11].

Muscular dystrophies themselves represent complex diseases and a diagnostic challenge. Therefore, we present a case of LGMD2B and the development of a rare dermatological condition, Morbihan disease.

## Case presentation

We present the case of a 38-year-old man from the State of Mexico. He worked as a warehouse assistant and driver's assistant for two years. No family history of consanguinity was reported. He had been diagnosed with systemic arterial hypertension, which was adequately controlled and treated. He presented with chronic sinusitis eight years prior to his current illness, with no evidence of symptomatic improvement despite treatment with daily intranasal mometasone for one year. He had a history of inguinal graft surgery, arthroscopy, and tendon reconstruction of the fourth finger of his right hand, all without complications.

**Figure 1:**
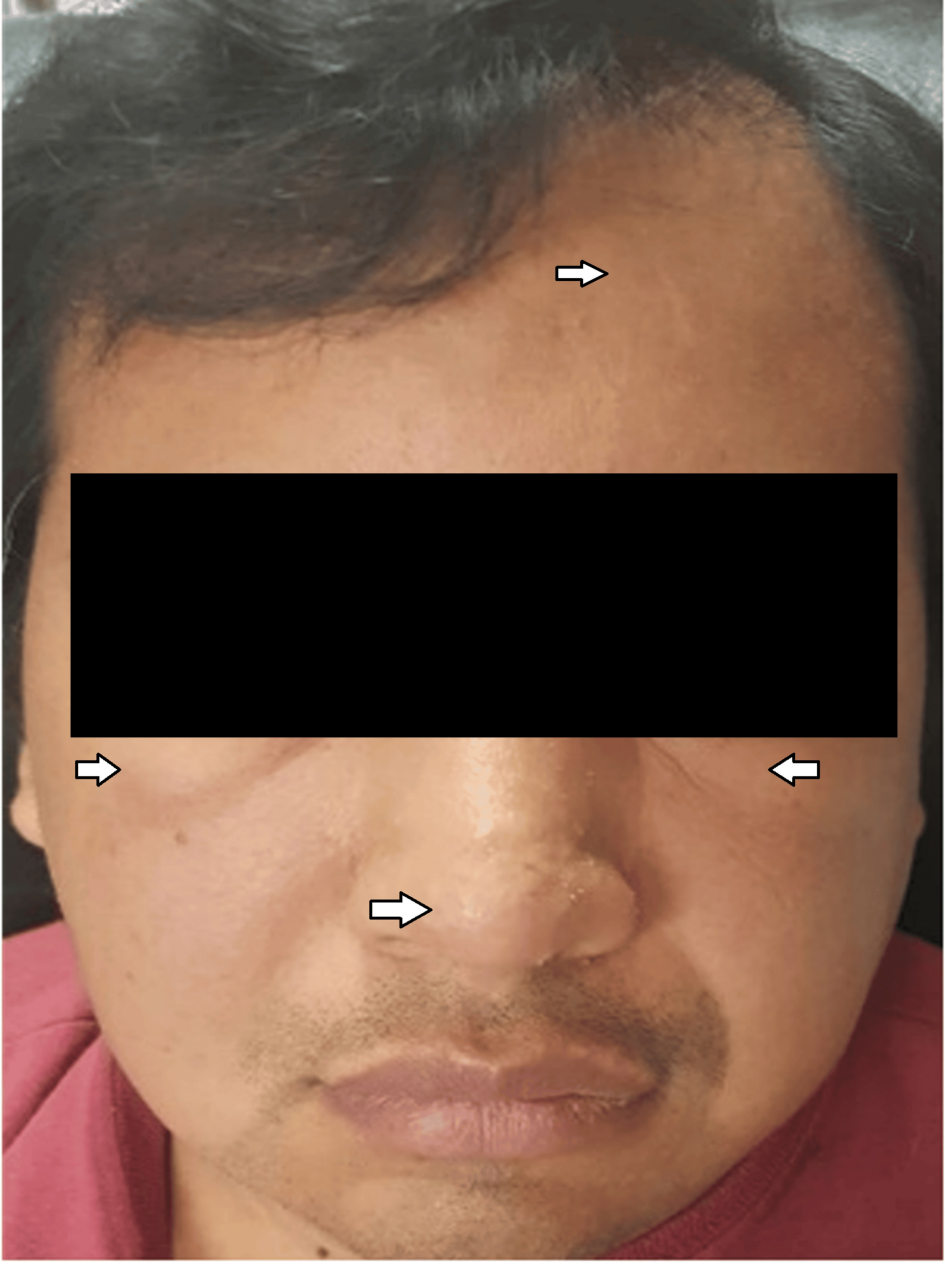
Papular and erythematous dermatoses located on the face, predominantly on the forehead, nose, and cheeks. Nontender facial edema, predominantly in the eyelids, nose, and forehead.

His illness began with facial edema, apparently gradual in onset and persistent for at least three months. At the same time, well-defined erythematous papular dermatoses and areas of erythema were identified on the face, predominantly on the forehead, nose, and cheeks (Figure 1). He was initially treated with daily antihistamines for 14 days, with no improvement in the lesions or facial edema. Therefore, treatment was started with epinastrin twice daily, montelukast once daily, and hydroxyzine three times daily for three months, with no improvement in the dermatosis or facial edema.

Subsequently, treatment was modified with prednisone 50 mg once daily for 14 days and danazol 100 mg daily for two years, with partial improvement of the lesions and persistent facial edema. Due to the lack of improvement despite the prescribed treatment and the chronicity of the symptoms, the patient was referred to a specialized medical discharge unit.

During questioning, the patient reported odynophagia, asthenia, and proximal weakness in the pelvic and thoracic extremities, accompanied by moderate muscle pain and cramps in both gastrocnemius muscles.

During the initial interview, the patient reported having suffered from a feverish syndrome lasting several days and occurring intermittently on several occasions throughout his childhood, accompanied by myalgia and arthralgia, with occasional difficulty walking, but which allowed him to engage in regular physical activity.

Physical examination revealed facial edema, predominantly in the orbit, papular lesions, and facial erythema. He had difficulty sitting and standing. His extremities were intact, and strength in various muscle groups was assessed according to the Medical Research Council (MRC) scale, with a score of 43 out of 60.

Due to the initial clinical presentation (facial edema, asthenia, muscle weakness, and myalgia), a thyroid profile was requested, which showed no evidence of abnormalities, thus ruling out thyroid disease.

Among the differential diagnoses, the possibility of an autoimmune disease was considered, with a high probability of dermatomyositis due to the presence of dermatosis and muscle weakness. Total CK levels were determined, which were only four times the upper limit of normal. Furthermore, LDH and CRP values ​​were within normal limits, which is not typical of inflammatory myopathy. Anti-HIV, anti-HBV, and anti-HCV antibodies were obtained, ruling out infectious causes.

Negative autoimmune markers reduced the likelihood of dermatomyositis, shifting the suspicion to hereditary myopathy (Table 1).

**Table 1:** Clinically relevant studies performed during the management of the disease. Only the elevation of CK is evident. *Value outside the normal range. TSH, thyroid-stimulating hormone; T3, triiodothyronine; T4, thyroxine; HCV, hepatitis C virus; HBc, hepatitis B core; HIV, human immunodeficiency virus; CA 19-9, carbohydrate antigen 19-9; CK, creatine kinase; LDH, lactate dehydrogenase; SSA (Ro), Sjögren’s-syndrome-related antigen A; SSB (La), Sjögren’s-syndrome-related antigen B; RNP, ribonucleoprotein; Scl 70, anti-topoisomerase I antibody; Jo 1, anti-histidyl tRNA synthetase antibody; ANA, antinuclear antibody

Parameter	Result	Units	Reference values
TSH	3.15	mUI/mL	0.4-4.5
Total T3	123	ng/dL	60-181
Free T4	1.13	ng/dL	0.93-1.7
Thyroglobulin	22.1	ng/dL	Oct 40
Anti-thyroglobulin antibody	<20.00	UI/mL	0-40
Anti-HCV antibody	0.04	-	≥1.0 reactive, 0.90-0.99 gray zone, ≤0.89 non-reactive
Anti-HBc antibody	0.05	-	<1.0 non-reactive, ≥1.0 reactive
Anti-HIV antibody	0.09	-	≥1.0 reactive, 0.90-0.99 gray zone, ≤0.89 non-reactive
Carcinoembryonic antigen	1	ng/mL	<5
CA 19-9	3.48	U/mL	<25
Alpha-fetoprotein	1.89	IU/mL	0.5-5.5
C-reactive protein	<3.44	mg/L	<3.0
Extractable nuclear antigen antibodies	-	-	-
Anti-SSA (Ro)	1.21	U	<20 negative, 20-39 low positive, 40-80 medium positive, >80 high positive
Anti-SSB (La)	1.18	U	<20 negative, 20-39 low positive, 40-80 medium positive, >80 high positive
Anti-RNP	1.48	U	<20 negative, 20-39 low positive, 40-80 medium positive, >80 high positive
Anti-Scl 70	1.35	U	<20 negative, 20-39 low positive, 40-80 medium positive, >80 high positive
Anti-Jo 1	1.28	U	<20 negative, 20-39 low positive, 40-80 medium positive, >80 high positive
Cryoglobulins	Negative	g/L	<0.05
Rheumatoid factor	<11.1	UI/mL	<20
Anti-DNA	Negative	UI/mL	<200
ANA	Negative	-	<1:80 negative
Blood chemistry	-	-	-
Glucose	108.43	mg/dL	70.0-105.0
Creatinine	0.98	mg/dL	0.5-1.0
Uric acid	7.26	mg/dL	3.5-7.2
Total cholesterol	232	mg/dL	140.0-220.0
Triglycerides	199	mg/dL	<150
Total CK	631.92*	U/L	50-150
LDH	211	U/L	230-460

Since no abnormalities were found in the aforementioned studies, and considering the clinical presentation with dermatological lesions and muscle weakness in a young patient, a paraneoplastic syndrome was intentionally sought. A computed tomography scan of the chest, abdomen, and pelvis was performed to search for tumors or lymph node clusters, with no findings using this method of study.

The persistent proximal muscle weakness progressively increased, as the patient went from walking independently to being wheelchair-bound over at least three months. Treatment with methylprednisolone 1 gram daily for five days was therefore initiated, with no improvement in symptoms.

A biopsy of the deltoid and gastrocnemius muscles was performed. A biopsy of the gastrocnemius muscle of the right lower extremity reported the presence of striated muscle without atrophy or chronic or acute inflammatory infiltrate in the endomysium or perimysium, and intramuscular blood vessels that showed mild congestion. An incisional biopsy of the left deltoid muscle revealed mild diffuse atrophy.

The study was then continued with EMG, which revealed data compatible with generalized myopathy with minimal membrane instability, even ruling out signs of neuropathy.

The patient received a daily dose of 75 mg of prednisone for one month, corresponding to a dose of 1 mg/kg/day, which was gradually tapered. He continued to experience myalgia, predominantly in the deltoids and both legs.

A new biopsy of the right quadriceps muscle was performed, which revealed moderate atrophy with a myopathic pattern. These results were complemented by electron microscopy, consistent with metabolic myopathy due to glycogen storage.

**Figure 2:**
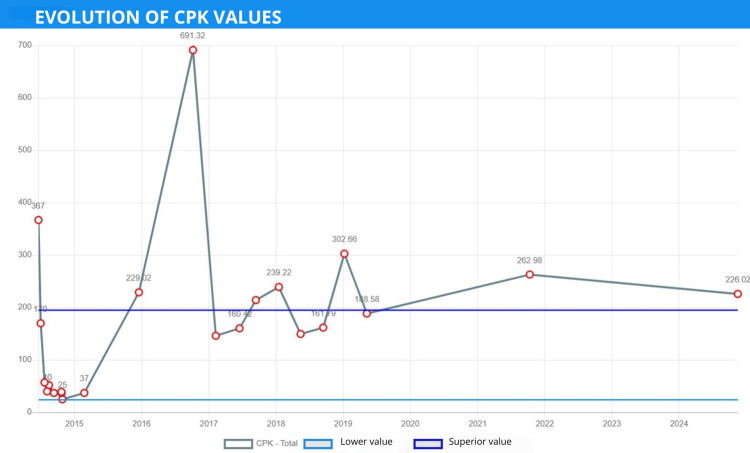
Evolution of serum CK levels. A higher elevation of up to 4 times the upper limit of normal was observed (normal range of 50-150 U/L). At this time, the patient was wheelchair-bound. CK, creatine kinase

The patient continued to experience this clinical syndrome, with no clear diagnosis to differentiate between an immunological or genetic problem. Clinically, the patient appeared to present only with muscular involvement, including weakness, fatigue, severe pain after physical activity, elevated CPK levels without the typical increase seen in dysferlinopathy (Figure 2), a myopathic pattern on electromyography, and an electron microscopy biopsy, suggesting glycogen storage.

Given the lack of response to treatment with high-dose systemic steroids and the exclusion of autoimmune causes, a molecular study for muscular dystrophy was requested. Molecular analysis of exon 15 of the DYSF gene revealed heterogeneity in the missense variant described as c.1382T>C; p. (Ile461Thr). The frequency of this variant is not recorded in the population databases ExAC, OMIM, dbSNP, or 1000 Genomes, and it is not recorded in the scientific literature. This variant showed the substitution of the nucleotide thiamine (T) for cytosine (T) in the DYSF gene, resulting in the substitution of an isoleucine (I) for threonine (T) at position 461 of the final protein.

It was classified as a variant of uncertain significance in the DYSF gene according to the American College of Medical Genetics (ACMG). Given the clinical data, the poor response to steroids, and the exclusion of other causes of muscle disease, it was concluded that this mutation demonstrated the presence of autosomal recessive LGMD2B.

Regarding the patient's facial edema and dermatosis, an association with muscle weakness was ruled out. Therefore, differential diagnoses were addressed, including acne rosacea, lichen planus, pityriasis varioliformis, polymorphic light eruption, erythema of the deep gyrus, indeterminate leprosy, and cutaneous tuberculosis.

A skin biopsy of the forehead was required, which revealed marked chronic perivascular and interstitial dermatitis, predominantly superficial, with granuloma formation in the reticular dermis, along with folliculitis and acute perifolliculitis with abscess. Based on these findings, Ziehl-Neelsen, periodic acid-Schiff, and Grocott stains were ordered, all of which were negative for microorganisms. It was decided to complement these studies with a biopsy culture, which was negative for mycobacteria. Due to the patient's clinical presentation and extensive laboratory studies that ruled out infectious and autoimmune diseases (e.g., lupus and dermatomyositis), thyroid disorders, and even neoplasia, a clinical and histopathological correlation was performed with the skin biopsy results, which supported the diagnosis of Morbihan disease (Table 2) [10,12,13].

**Table 2:** Differential characteristics of dermatomyositis and Morbihan disease. CK, creatine kinase; tRNA, transfer ribonucleic acid; SRP, signal recognition particle

	Dermatomyositis	Morbihan disease
History	Isolated presentation or in association with systemic autoimmune diseases, neoplasia, and rarely, infections or environmental exposure.	Symptoms of rosacea, acne, systemic conditions, allergic disease, or reaction.
Evolution	Skin lesions concomitant with the development of myositis. Subacute or chronic course.	Chronic skin lesions.
Topography	Bilateral and symmetrical eyelids. Mild edema. Bony prominences (metacarpophalangeal joints).	Upper two-thirds of the face (forehead, glabella, eyelids) and spread to cheeks. Bilateral and symmetrical.
Morphology	Papules, violaceous erythema. "Heliotrope rash." Hyperkeratosis.	Persistent solid edema, erythema, telangiectasias, papules, vesicles, nodules.
Laboratory	Elevated muscle enzymes (creatine kinase). Presence of myositis-specific autoantibodies (anti-tRNA synthetase, anti-SRP, and anti-Mi2). Tumor markers (in suspected neoplastic disease).	Generally unchanged.
Histology	Presence of mucin in the dermis.	Perivesicular and periadnexal infiltrate of lymphocytes, histiocytes, and neutrophils. Dermal edema, dilated lymphatic vessels, and clusters of mast cells. Peri- and intralymphatic granulomas.
Immunohistochemistry	Complement C5b-C9 staining.	D2-40 and CD31 staining confirm lymphatic involvement.
Treatment	Steroids, immunosuppressants (methotrexate, azathioprine), immunotherapy (rituximab).	Isotretinoin.

Morbihan disease is characterized by painless, nonpruritic facial edema and a history of rosacea or acne, which supports the diagnosis in the presented case. This disease is often considered a complication of these pathologies due to chronic inflammation and vasodilation. This is consistent with the presented case, as the patient's dermatological history included papular and erythematous lesions that accompanied the facial edema and persisted despite treatment. Upon completion of the diagnosis of Morbihan disease, the patient received treatment with isotretinoin 20 mg twice daily for eight months. The lesions and facial edema improved by up to 80% compared to the initial clinical picture.

The final diagnosis was Morbihan disease, which presented independently of LGMD2B.

## Discussion

The reported case was considered a diagnostic challenge, as the literature frequently mentions misdiagnoses in this type of presentation. It is common to confuse the diagnosis of inflammatory myopathy and initiate treatment for this condition in patients with muscular dystrophies, as described by Nguyen et al., where 25% of patients diagnosed with dysferlinopathies were misdiagnosed with polymyositis due to muscle biopsy findings revealing significant inflammatory changes and muscular infiltrates associated with rapid progression, with or without pain [4,14-16]. A previous case report by McMillan and Michaud describes a patient who, like our case, required multiple histological studies and was treated with steroids. In that study, they reported that at disease onset, perimysial and perivascular inflammation was observed in a first histological study and that a second biopsy showed changes compatible with established dystrophy, indicating that more than one histopathological study was required to conclude a diagnosis of muscular dystrophy and rule out an immune-mediated disease [9].

Furthermore, LGMD2B and polymyositis are very similar in both clinical symptoms and muscle histological staining. They share common features, including symmetric limb weakness, elevated CK, and electromyographic changes indicative of muscle injury. Therefore, immunohistochemistry and a genetic panel for LGMD using next-generation sequencing technology are particularly useful, as they can increase the sensitivity of LGMD2B diagnosis and play a key role in early treatment [17].

Disease onset is generally reported in patients over 18 years of age. In the present case, the age of onset was 38 years in a patient previously able to engage in physical activity. This has also been documented in the literature, as many patients actively participated in sports during adolescence before disease onset. In a systematic review by Audhya et al., LGMDR2 and LGMDR1 subtypes were reported as the most common among adult-onset patients, accounting for up to 43.4% [7].

According to the patient's clinical presentation, he reported increasing weakness and myalgia, along with biochemical alterations. The literature mentions that pain is not a typical feature; however, when present, it is usually mild, transient, and occurs after exertion. In the early stages, patients often present with an abnormal gait, difficulty walking or running, and, depending on progression, deterioration in ambulatory function, as seen in previous cases. CK levels are generally markedly elevated and can be 10 to 72 times higher than normal. In this case, the elevation was modest and not sustained. One limitation in identifying this pathology is that data on the disease's natural history are scarce, and there is a lack of studies describing larger clinical cohorts with long-term follow-up [18]. In this case, a heterozygous, autosomal recessive mutation in the DYSF gene was identified, described as NM_003494 (DYSF_v001): c.1382T>C; p.(Ile461Thr). This mutation is considered a variant of uncertain significance according to the ACMG guidelines. According to a study in India, autosomal recessive LGMD is the most prevalent form in the region, where nine suspected cases were evaluated and seven pathogenic mutations were identified. Among these, two variants of uncertain significance were noted: c.1347 C>A (p.N449K) and c.1376 A>G (p.Gln459Arg). The study also reported a 40% incidence of dysferlinopathies, with LGMD2 being the most common subgroup and a male-to-female ratio of 1.5:1 [3].

Regarding studies conducted in the Americas, a cohort of patients from Brazil, Argentina, Chile, Ecuador, and Colombia with clinical suspicion of LGMD underwent molecular diagnosis of muscular dystrophy using a genetic panel for LGMD via next-generation sequencing technology in 16% of patients; the same test was performed for our patient. The results showed that the most frequent diagnoses were LGMD2B related to dysferlin and LGMD2A related to calpain 3 [19].

In Latin America, a study was conducted on 2,103 patients, 53.7% of whom were men and 74% of whom were over 18 years of age, with the objective of identifying genetic variants. No patients were homozygous, compared to studies reported in other continents. The most common genetic variant was the DYSF gene (22%), with 37.9% in LGMD2B and 26.9% in LGMD1, which is relevant when comparing these data to the present case. Furthermore, the pathogenic variant found in our patient was not listed in this study [20].

In Mexico, a study confirmed a variant in the DYSF gene classified as pathogenic or likely pathogenic, thereby confirming dysferlinopathy in a Mexican family from an endogamous region. This mutation in the DYSF gene (c.792+2T>A) is homozygous, consistent with other worldwide studies, where consanguinity is one cause [8]. The present case did not report a history of endogamy or consanguinity, though this may be limited by incomplete parental lineage information. Our patient presented a heterozygous variant, which is a very rare form of presentation. However, a case report describes a 47-year-old man with clinical and biochemical characteristics similar to our patient. Like our case, a compound heterozygous inheritance pattern was evident, with two pathogenic variants detected in the NM_001130987.2 (DYSF) gene: c.2779del and c.4253G>A, showing that both parents and children were carriers of these variants [6].

These reported cases, including ours, contribute to identifying the DYSF gene variant as the most documented in Mexico and provide information on the disease's phenotype and genotype. However, studies with larger cohorts are needed to document DYSF gene mutations and variants in the Mexican population and to determine clinical presentation, disease evolution, and prognosis.

Regarding the Morbihan disease diagnosed in this patient, the dermatological lesions caused confusion and were initially associated with the patient's muscle weakness. This contributed to a suspected diagnosis of dermatomyositis, which required immunological and histopathological studies to reach the final diagnosis.

Histopathological findings included perivascular and periadnexal infiltrates of lymphocytes, histiocytes, and occasionally neutrophils [11]. The most characteristic findings were dermal edema, dilated lymphatic vessels, and clusters of mast cells.

These findings were documented in the histopathological study of our patient, which also highlighted granulomas, potentially causing lymphatic obstruction and vascular wall damage, as well as mast cell clusters. While epidermal granulomas are considered diagnostically indicative and correlate with disease stage, other infectious causes, such as tuberculosis or leprosy, must be ruled out [10]. In this case, skin biopsies required special stains for microorganisms and were sent for mycobacterial culture, both yielding negative results.

In Mexico, a retrospective study evaluated 15 patients diagnosed with Morbihan disease. Eighty-six point six percent were male, aged 19 to 65 years, and 33.3% had a granulomatous process on histopathology, similar to our patient.

Regarding the treatment our patient received for this disease, which was independent of LGMD, the use of isotretinoin as monotherapy or in combination with antihistamines, doxycycline, or minocycline has demonstrated satisfactory results within six to 12 months [13], consistent with our patient's progress.

## Conclusions

This case presented a difficult diagnosis due to its clinical characteristics and disease progression, but molecular sequencing using a molecular panel for muscular dystrophies and the clinical presentation were compatible with LGMD2B. No evidence of consanguinity was found in the familial history, so the heterozygous presentation is exceptional considering the inheritance pattern. Genetic sequencing panels are relevant for difficult-to-diagnose pathologies that can be misdiagnosed, leading to inappropriate treatment. Although LGMD has a progressive course and no treatment that modifies the disease's prognosis has yet been identified, therapeutic strategies aimed at improving the patient's quality of life can be considered. Routine molecular sequencing testing is not recommended; it is only recommended if there are clinical data compatible with the disease, in addition to the patient's family history.

Among the limitations found in this case report is the lack of a precise family history of one of the parents, given the importance of establishing consanguinity in this type of disease. Furthermore, during the patient's evaluation, the association of dermatological lesions and muscle weakness was considered, which resulted in independent diagnoses and could have been a factor in delaying the most appropriate treatment for Morbihan disease.
